# Individual Identification and Genetic Variation of Lions (*Panthera leo*) from Two Protected Areas in Nigeria

**DOI:** 10.1371/journal.pone.0084288

**Published:** 2014-01-10

**Authors:** Talatu Tende, Bengt Hansson, Ulf Ottosson, Mikael Åkesson, Staffan Bensch

**Affiliations:** 1 A.P. Leventis Ornithological Research Institute, Department of Zoology, University of Jos, Plateau State, Nigeria; 2 Department of Biology, Lund University, Lund, Sweden; 3 Grimsö Wildlife Research Station, Department of Ecology, Swedish University of Agricultural Sciences, Riddarhyttan, Sweden; University of Sydney, Australia

## Abstract

This survey was conducted in two protected areas in Nigeria to genetically identify individual lions and to determine the genetic variation within and between the populations. We used faecal sample DNA, a non-invasive alternative to the risky and laborious task of taking samples directly from the animals, often preceded by catching and immobilization. Data collection in Yankari Game Reserve (YGR) spanned through a period of five years (2008 –2012), whereas data in Kainji Lake National Park (KLNP) was gathered for a period of three years (2009, 2010 and 2012). We identified a minimum of eight individuals (2 males, 3 females, 3 unknown) from YGR and a minimum of ten individuals (7 males, 3 females) from KLNP. The two populations were found to be genetically distinct as shown by the relatively high fixation index (F_ST_  = 0.17) with each population exhibiting signs of inbreeding (YGR F_IS_  = 0.49, KLNP F_IS_  = 0.38). The genetic differentiation between the Yankari and Kainji lions is assumed to result from large spatial geographic distance and physical barriers reducing gene flow between these two remaining wild lion populations in Nigeria. To mitigate the probable inbreeding depression in the lion populations within Nigeria it might be important to transfer lions between parks or reserves or to reintroduce lions from the zoos back to the wild.

## Introduction

Human pressure, agricultural developments and industrialization are leading increasingly to the destruction, fragmentation and isolation of natural populations [1]. A consequence of these changes is loss of genetic variability [2] and increasing risk of extinction [3]–[5]. Their requirements for large home ranges, low fecundity and low numbers have made mammalian carnivores vulnerable to local extinction in fragmented habitats [6]. Mammals are often the dominant carnivores where they occupy the top position in the food chain, thereby serving as ecologically fundamental species for the stability of the ecosystem. Their decline or extinction may disrupt the food chains and alter the structure of ecological communities [7]. Sadly over the years, the activities of man have put on verge the future prospects of existence of many wild mammal species, especially large carnivores [8].

Lions (*Panthera leo*) once roamed most parts of Africa, Southern Europe, the Middle East and Asia [9]. Today they are only found in sub-Saharan Africa and at one locality in India where they are being increasingly restricted to protected areas and often in declining numbers [10]–[11]. In West Africa, lions are found only in protected areas such as national parks, game reserves and zoos. In Nigeria, the only protected areas known to still have wild lions are Yankari Game Reserve and Kainji-Lake National Park. The number of lions in these two isolates in West Africa has been poorly investigated. Population size estimate is an important biological parameter necessary for proper implementation of conservation measures [12]. Thus it is important to get adequate information on population size and connectivity between fragments of populations for proper conservation and management of a species [13]. Lions just like other large terrestrial carnivores are usually very difficult to count due to their elusive behavior and ability to cover large home ranges. An effort to conduct complete counts of a lion population is thus likely to be both organizationally difficult and time consuming [14].The alternative is to interpolate population sizes using different sampling strategies.

The use of DNA from non-invasive samples such as faeces, saliva, hairs or feathers for individual genetic tags can provide useful information for population monitoring as well as contributing with important genetic parameters [15]. Non-invasive sampling is widely used in genetic studies of elusive animal populations [16]–[18].This method is of prime importance in conservation genetics and behavioral ecology because it allows for genetic studies of wild animals without having to catch or even directly observe the animals under study [19], thereby reducing the possible amount of stress and harm inflicted on the animal. Various studies have employed the use of non-invasive sampling to identify individuals in a population, estimate population size [20], [12], [21], [16], [22], to monitor population sizes over time [23]–[24] and to also estimate the home range of individuals [20]. By studying the appropriate nuclear markers (most often microsatellites) analysis of non-invasive genetic samples (e.g. faeces) collected opportunistically from the field can provide individual identification, adequate information on population size, sex identification as well as genetic polymorphism within and between populations [25]. Knowledge of past events in a population and genetic structure is very important to assess the risk of extinction and chances of regional persistence of lion populations in Nigeria and elsewhere. Conservation of the genetic diversity of a species is important for preserving endangered wildlife [26]. This is because genetic diversity is the raw material for evolutionary change; that will allow the population to evolve in response to catastrophic changes such as new disease outbreak, pests, competition and predators.

In a pilot study carried out on the lions in Yankari Game Reserve (YGR from here on), Central North-Eastern Nigeria, between 2007–2008, we employed a population survey using genetic analysis of faecal DNA and showed the feasibility and reliability of the method [27]. In the present study, we employed the same method of faecal sample DNA analyses increasing the number of loci from two to nine to improve the precision of individual assignment. We also extended the survey to Kainji-Lake National Park (KLNP from here on), western Nigeria, which is a protected area that contains the most closely located and presumably only other Nigerian lion population to Yankari. We aimed to identify individual lions in YGR and KLNP and to determine the degree of genetic variability within and between these populations. Because the lions in the two parks have been isolated for several years, we expect them to be genetically differentiated. Also, the small population sizes suggest that there is a substantial level of inbreeding, which should be reflected by a positive inbreeding coefficient.

## Materials and Methods

### Ethics

The study was carried out with permission from the National Park service in the case of KLNP. In YGR, the A.P. Leventis Ornithological Research Institute had a Memorandum of Understanding (MoU) with the Bauchi State government to conduct any type of ecological research. Faeces are not part of the animal and as such faecal samples are not banned by CITES from being transported between countries.

### Study sites

**Figure 1:**
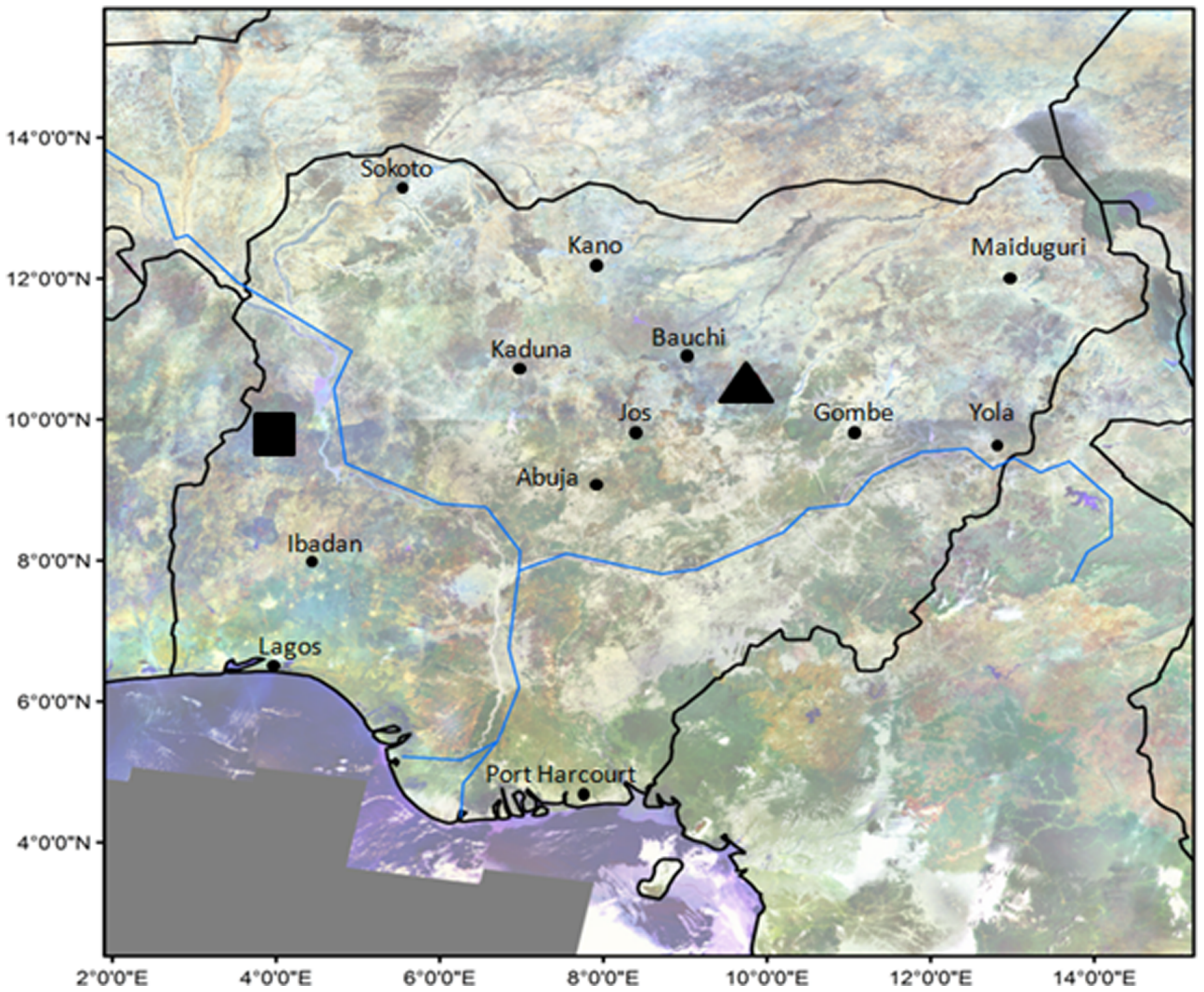
Map of Nigeria with some major cities and position of the two survey sites. Kainji-Lake National Park (KLNP) in black rectangle, Yankari Game Reserve (YGR) in black triangle.

Faecal samples were collected from two protected areas: Yankari Game Reserve (YGR) and Kainji-Lake National Park (KLNP) (Fig. 1).

The YGR is located in Central North-Eastern Nigeria with a landmass of 2,244 km^2^ (9° 50′N and 10° 30′E). Detail site description for YGR is contained in Tende et al. [27].

KLNP is located in the western part of Nigeria (10° 22′N 04° 33′E) and occupies a landmass of 5,340 km^2^. The vegetation is made up primarily of Guinea savanna woodland; common woodland species include *Terminalia macroptera* found along the Oli River which flows in the centre of the Park, *Detarium microcarpum* and *Borkea africana* woodland occupy about 70% of the Park area. *Isoberlinia tomentosa* woodland play vital role in providing shelter and cover for game. The mean annual rainfall is between 1000 and 1200 mm per year and occurs between April and October, with the highest peak of rain in September [28].

### Sample collection and extraction

A total of 3724 hours were spent sampling in YGR during data collection between 2008 and 2012, and 294 hours were spent sampling in KLNP in 2009, 2010 and 2012. The Global Positioning System (GPS) was used to record the position of each sample collected. Field methods are described in detail in Tende *et al*. [27]. All samples were preserved in 95% ethanol at room temperature, thereafter taken to Lund University, Sweden and kept in a freezer at −40°C before DNA extraction. DNA was extracted from all samples collected from YGR (n = 836) and KLNP (n = 93). DNA extraction was carried out using the QIAamp® DNA Stool Mini Kit (Qiagen).

Contamination of DNA during extraction or the PCR process can be a major problem when using non-invasive DNA; this was carefully taken care of by conforming to guidelines to avoid this through the use of a blank to control for contamination during the extraction and PCR processes.

### Mitochondrial DNA

A short (206 bp) portion of the mitochondrial cytochrome *b* gene was amplified and sequenced using PCR-based methods with locus specific primers to confirm samples that were from lions and to exclude a few cases of spotted hyena (*Crocuta crocuta*) or striped hyena (*Hyaena hyaena*). The primers LIHYF (5′- ATGACCAACATTCGAAAATCWC-3′) and LIHYR (5′-ATGTGGGTSACTGATGAG-3′) were designed to avoid amplification of human and ungulate DNA in general, in order to promote detection of the target species [27]. All amplifications were done using 2X Qiagen multiplex PCR kit in 10 µl reaction volume containing 5 µl Qiagen multiplex PCR buffer mix; 0.2 µM forward primer (Applied Biosystems), 0.2 µM reverse primer, 2.6 µl water and 2 µl of DNA extract with a hot start at 95°C for 15 minutes. PCR profiles consisted of 35 cycles as follows: 90°C for 30 seconds; annealing temperature of 52°C for 30 seconds with elongation period of 72°C for 30 seconds. A blank control (reagents only) from DNA extraction process was included in all PCRs to monitor for contamination. The results of the PCR were evaluated by electrophoresis using 2% agarose gels and GelRed™ (Biotium) staining. Positive samples were sequenced using LIHY forward primer (BigDye sequencing kit; Applied Biosystems) in an ABI Prism® 3100 capillary sequencer (Applied Biosystems).

The sequences were aligned against species reference sequences (Ascension numbers; EF437586.1, AJ809332.1 and EF107524.1) obtained from Genbank to determine species identity.

### Microsatellite amplification and genotyping

All lion samples were scored for allelic variability at nine polymorphic microsatellite primers (FCA001, FCA008, FCA026, FCA031, FCA045, FCA077, FCA126, FCA506 and FCA567 [29]. PCR amplifications were performed in 6 µl reactions containing 0.12 µl (concentration: 10 µM) dye-labelled (6-Fam, Hex or Ned) F-primer, 0.12 µl unlabelled R-primer (concentration: 10 µM), 3 µl of 2X Qiagen Master mix, 0.76 µl double distilled water and 2 µl DNA extract. PCRs were done in a GeneAmp 9700 thermocycler (Applied Biosystems) with the following profiles: 95°C for 15 min; 40 cycles at 94°C 30 s, 52°C 90 s, and 72°C 90 s; followed by an elongation period at 72°C for 10 min. Primers were multiplexed together in batches based on differences in fragment length and dye. The primer combinations are as follows: FCA001-FCA026-FCA031, FCA008-FCA045-FCA126, and FCA077-FCA506-FCA567. After amplification, alleles of the PCR products of the multiplex 3 loci, labelled with different dyes and of different lengths were separated using capillary electrophoresis in an ABI PRISM 3730 Genetic Analyzer (Applied Biosystems). Alleles were sized relative to GS500 ROX size standard and proof read and scored in Geneious vs. 5.6.6 (Biomatters).

### Molecular sexing

Sex of identified individuals were determined using X and Y specific primers (SMCX17 and DBY7); [30]. The primers have been designed to avoid non-target amplification [31]. PCR amplifications were performed in 6 µl reactions containing 0.12 µl F-primer (concentration: 10 µM), 0.12 µl R-primer (concentration: 10 µM), 3 µl of 2X Qiagen Master mixes, 0.76 µl double distilled water and 2 µl DNA extract). PCR profile conditions for the multiplex amplification of the SMCX17 and DBY7 fragments are as follows: An initial denaturation of 95°C for 15 min, 20 cycles of 94°C for 30 sec, 60°C for 40 sec (decreasing 0.5 per cycle) and 72°C for 90 sec, followed by 20 cycles of 94°C for 30 sec, 50°C for 40 sec and 72°C for 90 sec and a final elongation period of 72°C for 15 min. After amplification, 2.5 µl of each PCR product was evaluated using 2% agarose gel with GelRed™ (Biotium) staining and samples with one band were scored as females (XX) and samples with two bands as males (XY).

### Allelic drop-out

Most non-invasive sampling studies are often confronted with low quantity and quality DNA (i.e. degraded DNA) making it ideal to use PCR primers that amplify short DNA fragments [32], [33]. Highly degraded DNA may cause allelic drop-out which results in heterozygotes being typed as homozygotes due to failure of amplification of one of the alleles. To minimize or avoid genotype errors due to allelic drop out and false alleles three independent PCRs were performed for each locus and samples as suggested by Taberlet *et al*. [34]. No alleles were retained in further analysis unless they had been detected at least twice.

Partial genotypes are assigned in some individuals at some loci where only one allele could be observed more than once. Although there is a possibility that the partial genotypes might belong to a new individual, this method of assigning them with matching samples ensure conservative population estimation [35] by minimizing individuals created through erroneous, multi-locus genotypes (non-existent individuals). Only samples that amplified at between 4 and 9 loci were included in further analysis. According to Murphy *et al.* [36] a minimum of four loci are sufficient for accurate individual identification.

We scored samples as being from the same individual if they had been scored as the same sex, identical genotypes at ≥ four loci, and if the mismatching locus could be explained by allelic dropout. Such analyses allowed us to discern both the number of unique individuals as well as the number of “re-captured individuals”. Faeces with the same multilocus genotypes are considered as recaptures.

### Genetic Analysis

We used the identity analysis module in the program CERVUS [37] to identify individuals with unique genotypes within the data set. We also calculated number of alleles (K), allelic richness (A), observed (H_OBS_) and expected (H_EXP_) levels of heterozygosity, probability of observing identical genotypes by chance among unrelated samples (P_(ID)_) and probability of observing identical genotypes by chance among siblings (P_(ID)sibs_)from the microsatellite genotype data. The probability of identity, P_(ID)_, describes the probability that two individuals which are drawn at random from a population will have the same genotypes at multiple loci [25]. The software program CREATE [38] was used to create input files for use in the software program FSTAT v2.93 [39]. Inbreeding coefficient (F_IS_), population fixation index (F_ST_) and Jost's estimate of genetic differentiation (D_est_) were calculated using FSTAT and GenAIEx 6.5 [40]. Test for deviations from Hardy-Weinberg equilibrium exact test within populations was calculated based on 1000 randomisations, bootstrapping over loci at 95% CI. The nominal statistical significance value of 5/1000 was adjusted for multiple comparisons using the Bonferroni correction to minimize possible type I error. F_ST_ is used instead of R_ST_ [41] because it is considered to be a more reliable estimate of genetic differentiation when using small data set with less than 20 loci.

**Figure 2:**
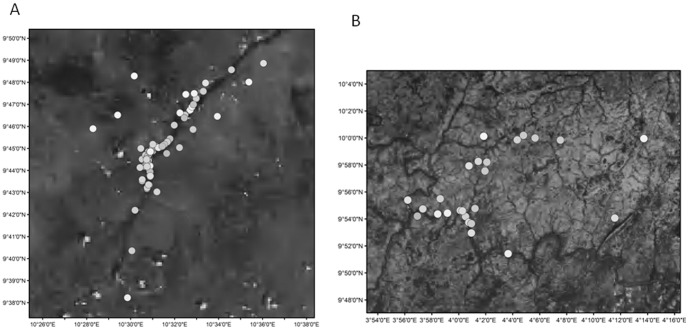
a: All genotypes sampled along the core area of Yankari Game Reserve. b: All genotypes sampled along the core area of Kainji-Lake National Park.

Waypoints of sampled genotypes were laid out on site maps to provide an overview of areas they were sampled during the survey in the two study sites (Fig. 2a and b). The software package Wild1[42] in R was used to obtain Minimum Convex Polygon of all individuals and afterwards the extension Xtools (version 9.2) in ArcView 3.3 was used to calculate the area covered by each individual in order to have an overview of their movement patterns. For individuals that were encountered more than three times, their home ranges were estimated by using the minimum convex polygon method. Since at least a minimum of three points are needed to make a polygon, this was computed only for individuals that were encountered at least three times during the survey.

## Results

Out of 929 samples collected from the two protected areas (YGR n = 836 and KLNP n = 93), 713 were successfully amplified for the partial cytochrome *b* (YGR, n = 625 and KLNP n = 88). The 216 samples that did not amplify at all were excluded from further analyses. Hence, the overall amplification success rate was about 77%. This is higher than the success rate of 40% (108 samples) that was obtained in the 2008 data from YGR [27]. There was no observed difference in the number of samples that successfully amplified in YGR and KLNP.

We determined species identity by aligning our sequences to Genbank reference sequences of the mitochondrial cytochrome *b* gene of lions, spotted hyena and striped hyena. Based on five informative nucleotide positions, 300 (42%) sequences out of the 713 samples matched with spotted hyena, while the remaining samples (n = 413) were found to match with lion.

To get individual identification, the 413 samples found to contain lion DNA from the two areas (YGR n = 352 and KLNP n = 61) were genotyped at the nine polymorphic microsatellites loci.

For the nine loci we obtained complete genotypes (at all 9 loci) for 115 samples from YGR and for 39 samples from KLNP. Partial genotypes (<9 loci) were obtained from additional 185 and 22 samples from Yankari and Kainji, respectively. Fifty two samples that amplified at less than four markers (i.e. 0–3 loci) were discarded from further analyses. Hence, the total number of samples with informative genotypes (≥4 loci) was 361.

In YGR the nine loci had a mean of 3.33 alleles with an expected heterozygosity (H_EXP_) of 0.63 (assuming Hardy-Weinberg equilibrium) and an observed heterozygosity (H_OBS_) of 0.32, whereas KLNP had a mean of 6.44 alleles with an expected heterozygosity (H_EXP_) of 0.82 and observed heterozygosity (H_OBS_) of 0.51 (Table 1). The lion populations in Kainji and Yankari showed significant signs of inbreeding with F_IS_  = 0.49 in YGR and F_IS_  = 0.38 in KLNP (p<0.001, Table 1). The two populations were found to be genetically differentiated with F_ST_  = 0.17 and D_est_  = 0.65 (bootstrapping over loci the ±95% CI of F_ST_ was between 0.10–0.23, p = 0.004).

**Table 1:** Summary of genetic diversity; number of alleles (K), allelic richness (A), sample size (N), Observed and Expected heterozygosity (H_OBS_ & H_EXP_) and inbreeding coefficient (F_IS_) in the two populations over the years.

Locus	N	K	A	H_OBS_	H_EXP_	F_IS_	N	K	A	H_OBS_	H_EXP_	F_IS_
FCA001	8	4	3.61	0.50	0.60	0.29	5	4	4.00	0.25	0.82	0.72
FCA008	5	3	2.77	0.20	0.51	0.63	10	6	4.06	0.40	0.77	0.50
FCA026	8	3	2.88	0.37	0.62	0.41	10	9	5.66	0.70	0.90	0.23
FCA031	4	2	2.00	0.50	0.65	0.14	10	6	4.71	0.20	0.83	0.76
FCA045	8	2	2.00	0.00	0.53	1.00	8	6	4.49	0.57	0.79	0.29
FCA077	8	6	4.37	0.70	0.80	0.07	8	6	4.61	0.83	0.87	−0.05
FCA126	8	2	2.00	0.00	0.53	1.00	8	5	3.80	0.37	0.71	0.49
FCA506	7	3	2.44	0.14	0.69	0.86	8	7	5.05	0.50	0.85	0.43
FCA567	8	5	3.93	0.50	0.72	0.32	10	9	5.43	0.80	0.87	0.08
**Mean**	-	3.33	2.88	0.32	0.63	0.49	-	6.44	4.64	0.51	0.82	0.38

Yankari Game Reserve (N = 8) Kainji-Lake National Park (N = 10)

A total of eight individuals (2 males, 3 females, 3 unknown) were identified in YGR if we assume that allelic drop-outs had affected the scored genotypes (Table 2), while ten individuals were identified in KLNP (7males, 3 females, Table 3). The 3 individuals in YGR whose sex could not be determined molecularly were due to shortage of DNA template. Based on the observed allele frequencies there was a low probability of observing identical genotypes P_(ID)_ from two randomly sampled individuals from the same population in both YGR (P_(ID)_  = 0.00000213, P_(ID)sibs_  = 0.00259) and KLNP(P_(ID)_ = 0.00000000150, P_(ID)sibs_  = 0.000188). The mean polymorphic information content (PIC) values in both YGR (0.50) and KLNP (0.73) were high.

**Table 2:** Identified individuals in Yankari Game Reserve.

SampleID	IndvID	Sex	#times sampled	FCA001	FCA026	FCA031	FCA567	FCA007	FCA506	FCA008	FCA045	FCA126
YGR321	Y#1	-	4	129/155	128/128	***/***	85/85	135/144	244/244	***/***	125/125	124/124
YGR242	Y#2	F	107	127/127	128/137	242/244	94/96	146/153	214/214	117/117	127/127	124/124
YGR211	Y#3	-	5	129/155	128/128	***/***	85/85	130/135	244/244	***/***	125/125	124/124
YGRB1	Y#4	M	6	127/129	128/130	***/***	85/85	135/141	244/244	124/124	125/125	124/124
YGRN1	Y#5	M	21	127/127	128/128	242/242	94/105	141/153	191/191	117/117	127/127	127/127
YGR13	Y#6	F	14	127/127	128/137	244/244	96/105	141/153	191/214	117/117	127/127	127/127
YGR56	Y#7	F	1	137/155	130/130	***/***	85/85	135/135	***/***	***/***	125/125	124/124
YGR7	Y#8	-	1	127/127	137/137	242/244	94/107	153/153	214/214	117/117	127/127	***/***

Shown are; Sample identity (SampleID), individual identity (IndvID), sex and allelic length at the nine scored loci, *** (indicates missing data).

Microsatellite Loci

**Table 3:** Identified individuals in Kainji-Lake National Park.

Microsatellite loci												
SampleID	IndvID	Sex	#times sampled	FCA001	FCA026	FCA031	FCA567	FCA077	FCA506	FCA008	FCA045	FCA126
**KLNP13**	K#1	M	4	127/129	130/137	234/246	98/103	148/153	193/***	129/133	127/127	***/***
**KLNP18**	K#2	M	1	***/***	122/130	224/224	78/78	***/***	229/229	129/129	***/***	129/191
**KLNP19**	K#3	M	1	***/***	139/141	238/238	81/85	137/141	203/214	125/125	139/153	133/151
**KLNP4**	K#4	M	3	***/***	134/139	238/252	83/85	137/139	195/203	119/133	139/139	139/139
**KL26**	K#5	M	1	145/145	141/141	238/238	83/85	137/153	195/195	125/125	139/139	133/133
**KL18**	K#6	F	7	***/***	137/137	246/246	103/105	148/151	191/191	133/133	145/149	131/139
**KL11**	K#7	F	7	153/153	134/141	238/238	83/85	137/139	195/195	125/125	139/139	133/133
**KL9**	K#8	M	1	129/129	128/130	234/234	98/111	148/153	85/191	133/133	***/***	139/139
**KL33**	K#9	F	1	153/153	134/139	***/***	85/85	153/153	193/208	117/133	***/***	133/133
**K22**	K#10	M	1	145/153	144/148	244/244	83/94	137/***	197/203	127/129	149/153	133/***

Shown are: Sample identity (SampleID, individual identity (IndvID), sex and allelic length at the nine scored loci, *** (indicates missing data).

**Figure 3:**
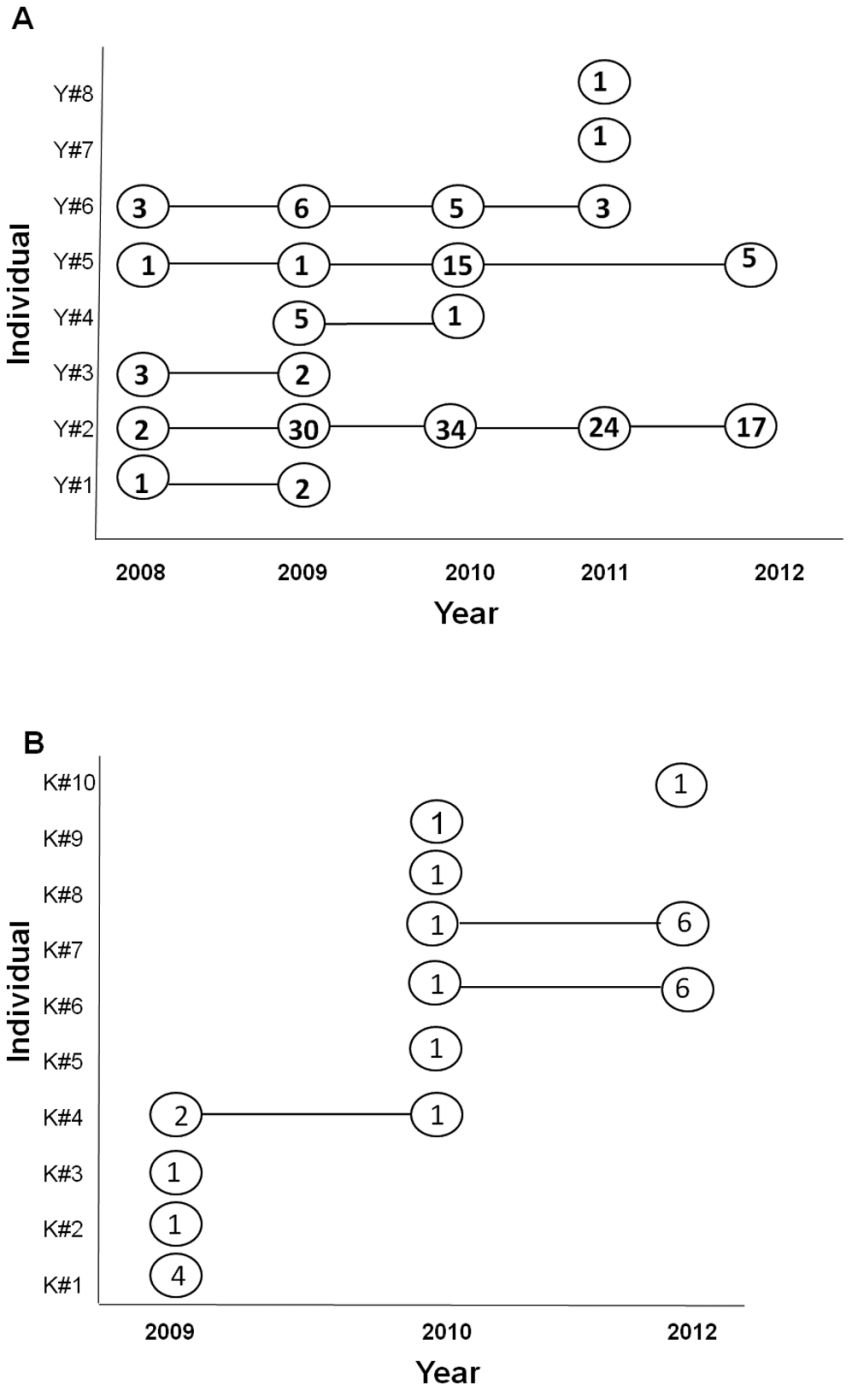
a: Sampling frequency per individual and year in Yankari Game Reserve. b: Sampling frequency per individual and year in Kainji-Lake National Park.

Within both survey areas, some of the identified individual genotypes were encountered in more than one year (YGR; n = 6, KLNP; n = 3), while some were encountered several times within a year and others were encountered only once or twice. In YGR individual Y#2 (female) and Y#5 (male) were observed to be present in the population all through the five year period. Individual Y#6 (female) was sampled from the beginning of the survey until 2011 (Fig. 3a). The combined genotypes of Y#2 and Y#5 are compatible with the hypothesis that the genotypes of Y#6 and Y#8 are their offspring (Table 2).

In both YGR and KLNP all individuals were sampled within the core area of the reserve (Fig. 2a and b). The home range analysis using the minimum convex polygon method gave an average home range of 11.91 km^2^, ±2.1 SD (1.71– 47.62 km^2^) in YGR (Appendix S1) and 26.75 km^2^ ± 1 SD (14.63– 39.37 km^2^) in KLNP (Appendix S2).

## Discussion

### Number of individuals

Based on the encounter of unique genotypes during the different survey years, a minimum of eight individuals was identified in YGR between 2008 and 2012, while ten individuals were identified in KLNP between 2009, 2010 and 2012. During the course of the survey there has been at least one observation of new cubs in the field in 2010 in YGR. Recently, there have also been sightings (between 8^th^ and 12^th^ March 2013) of 2 adult females with 3 cubs, 2 adult females without any cubs, 1 adult male with a mane together with 3 cubs in YGR. The sightings of these adults within YGR (1 male and 4 females, or 1 male and 2 females, (if we assume that it was the same females that were seen at different times) contradict the findings of Wildlife Conservation Society (WCS) who has reported, through call-up stations, that only two adult lions exist within the reserve [43]. These sightings are also confirmed by our molecular findings (Table 2), where females Y#2 and Y#6 and male Y#5 have been present most parts of the survey periods. Six of the eight identified individuals have been sampled multiple times (3-107 times) during the survey (Fig 3a). Our estimate gives the minimum number of lions that exist in both YGR and KLNP. Since several individuals have been encountered only once, especially in KLNP, the true number is potentially higher. The low estimates of probability of identity (P_(ID)_ and P_(ID)sibs_) obtained in our study both in YGR and KLNP gives confidence to the identity of individuals identified in these two survey areas and support the uniqueness of the identified individuals. Waits *et al.* [44] suggested that these values should be between P_(ID)_  = 0.01 – 0.0001 in studies estimating population size.

Despite the fact that there were fewer visits and also few samples collected from KLNP, we observed more individuals as well as a higher genetic diversity than in YGR. This suggests that there are and have been more individuals in the recent history in KLNP, which have contributed to a larger effective population size and a higher genetic diversity. Alternatively, the high diversity in KLNP may have resulted from connecting with gene flow with lions in the W-Arli-Pendjari complex, a vast protected area in Benin, Burkina Faso and Niger.

### Comparison with previous study

A number of studies conducted on the natural populations of African lions have been carried out mostly in Eastern, Central and Southern Africa and in India. These studies have employed opportunistic field observation and/or genetic data to understand behavioral, social and breeding structure of the lions [45]–[53]. Researchers have incorporated molecular methods to understand the genetic status of the lions in their natural populations (c.f. [50], [54]), characterize their evolutionary history, as well as to study disease outbreaks [55]–[58]. Our study has employed non-invasive samples with aid of molecular techniques to make available information on the population size as well as the genetic status of the remaining relict wild lion populations in Nigeria. The method does not have any negative impact on the study species.

The number of individuals estimated in YGR in the present study is lower than the number recorded during the pilot survey conducted by Tende *et al.* [27] where eleven individuals were identified from two microsatellite loci. This difference could be due to either a disappearance from the population of some individuals due to natural deaths or to the activities of poachers within the reserve, or it could be an overestimation in our previous study due to the low number of loci used. The use of polyacrylamide gel to genotype individuals during our pilot study [27] could possibly also have biased our estimation due to bad gels where stutter bands might have been typed as true bands.

During the course of the laboratory analysis and efforts to optimize different primers for the study, we ran short of DNA template from most of the extracted samples from the first pilot study in YGR. Hence, not all samples analyzed in our 2008/2009 pilot survey could be rerun on the new microsatellite primers used. However, in some samples where we still had DNA template it was observed that certain individuals have persisted in the population throughout the survey years.

The higher amplification success rate attained in this study as compared to 2007/2008 could be attributed to the new PCR kit/technique (Qiagen multiplex PCR kit) that was employed and also we concentrated our sample efforts on fairly fresh faeces. The shorter time the faeces are left out in the environment, the less degradation of DNA and this is especially true in tropical environments where high temperature and UV radiation, can cause fast degradation of DNA [59].

### Home range estimates

Many of the identified individual genotypes were encountered several times during the study. Lions are known to use large home ranges to satisfy energetic demands, but this can be limited where required resources have clumped distribution [50], [60]. This is the case during the dry season, for both study sites, when game concentrates close to rivers - Gaji River in YGR and Oli River in KLNP. Both rivers run through the core areas of the respective reserve/park and offer lush vegetation for prey species, as well as cover for the lions to rest and also stalk. It is also the main source of drinking water both for the predators and the prey. A similar survey by Spong *et al.* [50] to determine space use by lions in Selous Game Reserve, Tanzania, showed the most intensively used area to be small within the reserve and averaged only 11.7 km^2^, which is consistent with our finding in YGR where individuals were sampled mostly within the core areas which are intensively used and this averaged at 11.9 km^2^ (SD: ±2.1). They found out that prides often had close relatives in neighbouring prides but in areas with high prey abundance the home ranges between individuals, irrespective of relatedness tended to overlap more [50]. A survey by Lehmann *et al.* [60] on home range utilization of lions in Karongwe Game Reserve, South Africa, showed the home range used by a pride ranged between (10.3 – 64.4 km^2^) and for a single male ranged between (5.0 – 56.3 km^2^). Their findings about home range size are slightly larger than what we have obtained in our study (YGR: 1.71– 47.62 km^2^; KLNP: 14.63–39.37 km^2^). In our study a female (Y#2) was observed to have the largest home range (47.52 km^2^), this might probably be due to the need to hunt and feed the cubs and the male. This female has also been observed to persist in the population since the onset of this survey in 2007/2008. Both of the above studies [50], [60] employed the use of telemetry. Our study has shown that genotype data also can be used to determine the home range of lions, and possibly other mammals, enhancing sample size and reducing disturbance to the animal under study.

### Inbreeding

The populations of lions in both KLNP and YGR exhibit significant signs of inbreeding. This is not surprising given their small population sizes. The inbreeding level found in YGR in the present study is in line with our pilot study conducted in 2008 [27] when the inbreeding coefficient was estimated to be 0.21, whereas in this present survey the value was found to be 0.49 (Table 1). The inbreeding levels in both YGR (0.49) and KLNP (0.38) are high and comparable to what has been recorded in some other carnivore species. For instance the estimated inbreeding coefficient in the Scandinavian wolf (*Canis lupus*) population was up to 0.41 [61], [62], [17] before the population was genetically rescued by one immigrant from Finland [63]. The arrival of this immigrant into the Scandinavian wolf population provided the possibility to avoid inbreeding, decrease the risk of inbreeding depression and cause population growth. High inbreeding coefficient reaching up to 0.37 has also been recorded in the brown bear (*Ursus arctos*) [64]. Inbreeding and subsequent negative effects of inbreeding have been reported in the lions in Ngorongoro Crater in Tanzania [65], [48]. The number of alleles at a set of microsatellite loci for the Etosha lion population (A = 4.6) reported by Lyke et al. [53], and that reported by Antunes et al. [58] (A = 4.4) is similar to that detected in KLNP (A = 4.6), but higher than in YGR (A = 2.8), where Lyke et al. [53] detected no sign of inbreeding in the Etosha population (F_IS_  = 0.03). The inbreeding coefficient recorded in our study is higher than what has been recorded in the lion population in Etosha National Park in Namibia. Some studies (e.g., [4], [66]–[68]) have shown that if populations remain small and isolated for many generations they are bound to face increased inbreeding and gradual erosion of genetic variability. The lion populations in Nigeria are small, isolated and restricted to two protected areas (YGR and KLNP) where their populations may be declining. This is of course a threat to the long-term survival of these populations and to the lion population throughout West Africa [69].

Low genetic variability has been reported in lions in Ngorongoro Crater in Tanzania [48], [55], [70], [71]. Although there are recent observations of cubs within one of our study systems (YGR) the genetic status of this population as shown by our study is poor. This might affect individual survival and reproductive success in the long term if proper measures are not implemented. O'Brien [72] has observed that there is a strong correlation between genetic variation and reproductive parameters in lions. This means that with time the fitness of individuals within the lion populations in Kainji and Yankari will decline due to accumulation and expression of recessive and detrimental alleles with subsequent inbreeding depression, which might consequently drive the population to extinction.

### Population structure

The YGR and KLNP populations were found to be genetically differentiated (F_ST_  = 0.17, D_est_  = 0.65). This is not surprising due to the fact that these populations are small and isolated from each other; about 1000 km apart and they are separated by dispersal barriers including highways, agricultural landscapes and cities. Without any corridor for dispersal, isolation is expected to build up the observed pattern of allelic differentiation between the two populations. Moreover, high human and livestock densities characterize most of the surroundings of these protected areas, which can increase mortality risk of cubs and “possible dispersers” because of overlap with human habitations and livestock. All these factors may act as barriers to gene flow between the two populations and possible populations in neighboring countries. Studies have shown that geographical and environmental features can influence gene flow and genetic variation within and between populations [73], [74]. Studies carried out in California on mountain lion (*Puma concolor*), and coyote (*Canis latrans*) and bobcat (*Lynx rufus*) to assess the level of gene flow and differentiation in these carnivores showed how anthropogenic obstacles such as roads, agriculture and urbanization were great barriers to dispersal and gene flow [75], [76]. It was shown how these barriers imposed artificial home range boundaries on territorial carnivores thereby decreasing genetically effective migration, which leads to population differentiation.

A study carried out by Antunes *et al*. [58] to assess genetic variation from 357 lions from most of its current range in Africa and Asia using microsatellite data revealed significant population structure (F_ST_  = 0.03–0.79) within East-African lion populations despite the low geographic distance within them. Their finding is in accordance with our finding about the two lion populations in Nigeria (F_st_ = 0.17).

The existence and maintenance of genetic diversity and good connectivity between subpopulations are essential factors for long-term viability of a population [77], which should serve as the primary target of any acceptable conservation management program. Genetic diversity is the raw material needed in order to evolve the ability to cope with environmental challenges such as disease outbreak and parasites [78].

### Conservation genetics and management

The two lion populations are small and genetically different with signs of inbreeding within each population. This might elevate their risk of extinction in the face of sudden environmental catastrophes in the future. It has been shown that small populations are often faced with a higher risk of extinction from environmental catastrophes [3], [4], [5], [79]–[83] than large interconnected populations [84], [77], [85].

The main aim of a conservation genetic approach is to maintain diversity within and between populations so as to enhance the evolutionary potential of the population to cope with environmental changes. Laboratory and translocation experiments have indicated that small and inbred populations can be rescued by contribution of minimal number of immigrants [86]–[88], [62], [63]. This can help to decrease inbreeding and inbreeding depression [86], [89], and bring about profound changes in genetic structures [90], [91], [63]. Thus to mitigate the inbreeding condition and subsequent probable inbreeding depression in the lion population within Nigeria it might be important to transfer lions between parks or reserves or to reintroduce lions from the zoo back to the wild. This is necessary in order to enhance their genetic diversity for long term survival and reproduction. The bringing together of genetically dissimilar mates, hybrid vigor [89]–[91] can be advantageous because it will enhance reproductive success and also fitness [92]–[93], [68]. However, it would be recommended that lions that are genetically similar to the receiving population should be preferably used in any translocation program in order to avoid introduction of potentially non-locally adapted genotypes.

## Supporting Information

Appendix S1 **Estimated home ranges for some individuals in Yankari Game Reserve.**
(TIF)

Appendix S2 **Estimated home ranges for some individuals in Kainji-Lake National Park.**
(TIF)
