# Supplementary figures and images for: Individual Identification and Genetic Variation of Lions (Panthera leo) from Two Protected Areas in Nigeria
Source: PLoS One. 2014 Jan 10;9(1):e84288. doi: 10.1371/journal.pone.0084288 (PMC3888380; doi:10.1371/journal.pone.0084288)

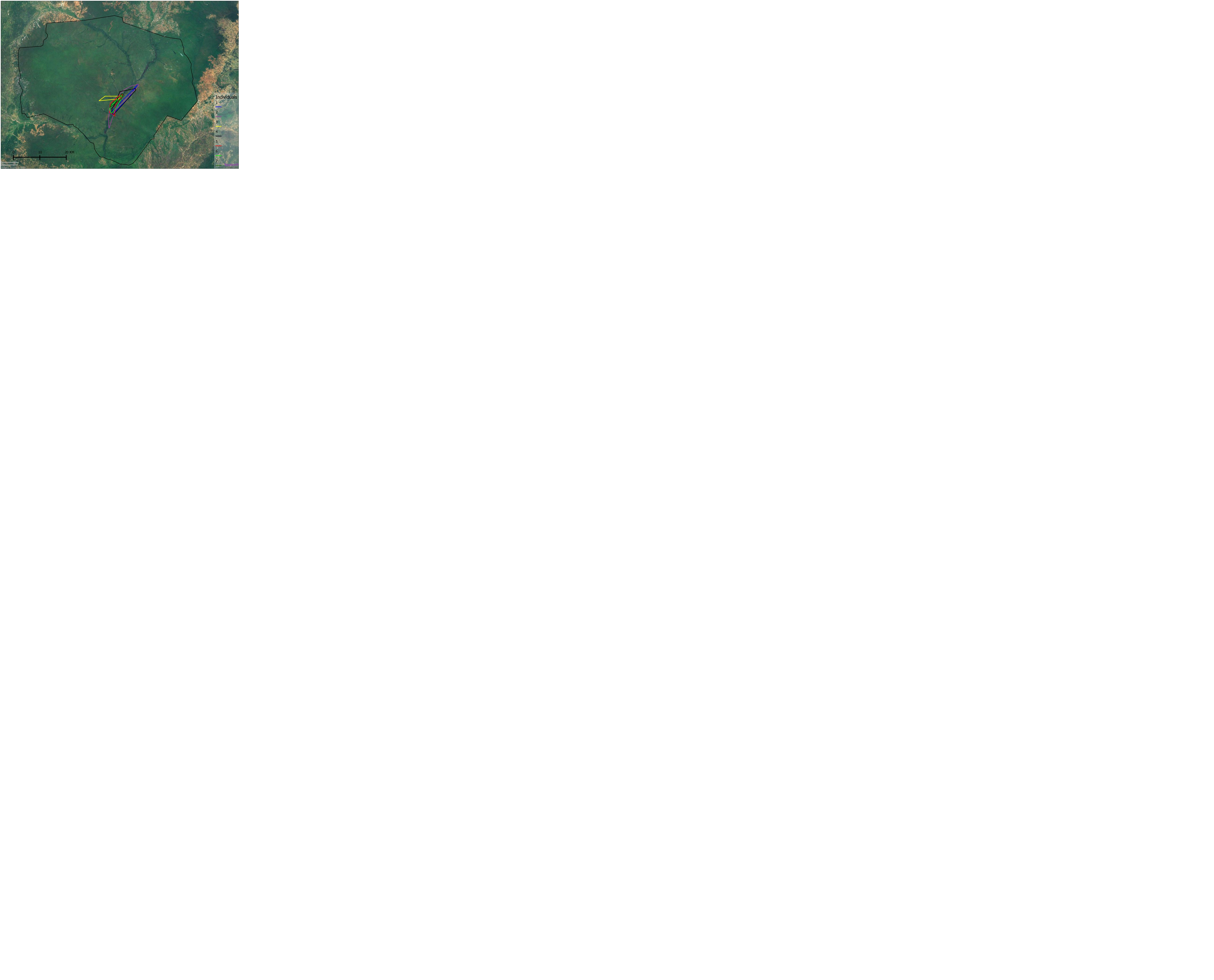

Supplement: Appendix S1 — Estimated home ranges for some individuals in Yankari Game Reserve. (TIF) [file pone.0084288.s001.tif]

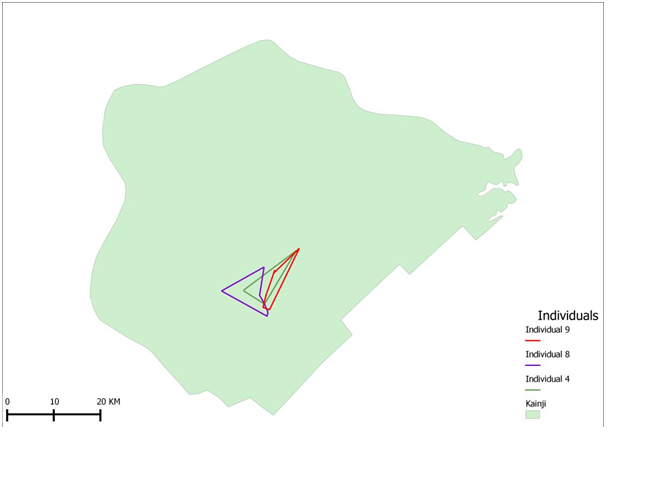

Supplement: Appendix S2 — Estimated home ranges for some individuals in Kainji-Lake National Park. (TIF) [file pone.0084288.s002.tif]
